# Diverging sex ratios in dioecious Proteaceae are exacerbated by anthropogenic disruptions to the fire cycle

**DOI:** 10.1093/aob/mcaf312

**Published:** 2025-12-09

**Authors:** Sarah F Visser, Seth D Musker, Michael D Cramer

**Affiliations:** Department of Biological Sciences, University of Cape Town, Rondebosch 7701, Cape Town, South Africa; Department of Biological Sciences, University of Cape Town, Rondebosch 7701, Cape Town, South Africa; Department of Biological Sciences, University of Cape Town, Rondebosch 7701, Cape Town, South Africa

**Keywords:** Anthropogenic, dioecious, diverging secondary sex ratios, fire suppression, Proteaceae, *Leucadendron* sp, *Aulax* sp, reproductive costs

## Abstract

**Background and Aims:**

Secondary sex ratios in dioecious plant species often deviate from the expected 1:1 primary male-to-female ratio due to differential survival rates. Such deviations in life-history strategies, along with diverging reproductive trade-offs, have been used for assessing reproductive costs in plants. In the fire-prone fynbos biome, previous studies on sex ratios and reproductive costs in dioecious Proteaceae have produced conflicting results, warranting further investigation. We examined whether obligate reseeding serotinous *Leucadendron* and *Aulax* species (Proteaceae) experience higher reproductive cost in males, females, or both equally.

**Methods:**

We analysed sex ratios across populations of varying ages and assessed individual health through canopy cover scores. In addition, we conducted nutrient analysis to quantify allocation to vegetative versus reproductive structures.

**Key Results:**

We found no evidence that primary sex ratios differ from 1:1, but clear evidence of secondary sex ratios becoming increasingly male-biased with age. Predictions indicated that a typical 30-year-old population would have a sex ratio of 0.67 (95 % CI 0.52, 0.81), corresponding to twice as many males as females. In older, more male-biased populations, females exhibited lower health scores. While total nutrient content did not differ between the sexes, females allocated a greater proportion of total nutrients to their reproductive cone structures.

**Conclusions:**

Our results suggest that females experience higher reproductive costs, which contribute to increased female mortality over time, resulting in male-biased sex ratios in older populations. Anthropogenic fire suppression likely contributes to this trend by allowing populations to survive beyond the natural fire-return interval for fynbos vegetation. These altered population dynamics could undermine long-term population viability and ecosystem stability in fire-adapted dioecious Proteaceae of the fynbos.

## INTRODUCTION

In dioecious angiosperm species, where male and female structures are on separate individuals, total resource allocation to reproductive functions such as pollinator attraction in males or seed production in females can differ between the sexes (Delph, 1999). The ‘cost of reproduction’ is defined as a reduction in future reproductive output due to investments made in current reproduction (Delph, 1999; Obeso, 2004; Dorken *et al.*, 2025). When one sex has a higher reproductive effort than the other, resulting in adverse effects on growth, survival or fecundity, that sex is said to experience a higher cost of reproduction (Tuomi *et al.*, 1983; Obeso, 2002; Bazzaz *et al.*, 2005; Dorken *et al.*, 2025).The primary sex ratio refers to the ratio of male to female individuals at the time of sex determination (e.g. during seed or seedling development), whereas the secondary sex ratio reflects this ratio among mature, flowering individuals after differential growth or survival (de Jong and Klinkhamer, 2002). Dioecious angiosperms predominantly exhibit a 1:1 primary sex ratio, consistent with Fisher’s principle, whereby a balanced ratio is maintained through negative frequency-dependent selection on the rare sex (Fisher, 1930; Hamilton, 1967). On the other hand, sex-biased mortality may result in persistent sex differences in lifespan that manifest as diverging secondary sex ratios (Marais and Lemaître, 2022). Although secondary sex ratios that diverge from the primary ratio often indicate differing costs of reproduction in dioecious angiosperms (Delph, 1999; Obeso, 2002), sex-specific differences that are not necessarily related to reproductive investment can also shift secondary sex ratios away from primary ratios (Field *et al.*, 2013; Liu *et al.*, 2021). Regardless of their underlying cause, biased secondary sex ratios may have important implications for the viability of future populations by limiting seed recruitment. For example, a reduction in the proportion of either sex may reduce overall reproductive success and thus reduce population size and genetic diversity in subsequent generations (Rosche *et al.*, 2018). Long term, persistent deviations in secondary sex ratios are therefore potentially unsustainable, and a sign of altered population dynamics.

The South African Cape Floristic Region (CFR) is home to the fire-adapted fynbos biome, which is a resource-constrained system characterized by limited nutrients, particularly phosphate (Cramer *et al.*, 2014). Fire plays a crucial role in this ecosystem by recycling these limited nutrients back into the soil (Verboom *et al.*, 2024). Within fynbos’s characteristic Proteaceae family are the genera *Leucadendron* and *Aulax*, both of which are dioecious and frequently exhibit extreme phenotypic sexual dimorphism (Barker *et al.*, 2004). Many of the species in both genera are serotinous, retaining seeds in cone-like inflorescent structures (hereafter ‘cones’) that open and release seeds en masse after fire (Barker *et al.*, 2004). The predominance of post-fire recruitment and limited seed dispersal results in populations within a burned area being dominated by plants of similar ages (Midgley, 1987). This phenomenon of a single age cohort only applies to non-resprouting, obligate seeding species, as resprouting individuals can survive through multiple fires and therefore can be older than the surrounding vegetation. The fire-adapted life history of dioecious, obligate reseeding Proteaceae therefore mitigates the usual challenges of quantifying age-related deviations in sex ratios that arise from staggered recruitment in other systems.

Fynbos vegetation is thought to experience natural fire intervals of ∼12–15 years (Wilgen *et al.*, 1994). Anthropogenic influences have disrupted these natural fire intervals (Slingsby *et al.*, 2020), impacting fynbos biodiversity (Vlok and Yeaton, 1999; Verboom *et al.*, 2024) and potentially compromising the seed bank either by preventing seed accumulation under too-frequent fire regimes, or by scorching seeds during excessively hot fires resulting from biomass accumulation under infrequent fire regimes (Kraaij and van Wilgen, 2014). Therefore, fire suppression may cause individuals within populations to attain ages that exceed those typically induced by the natural fire regime under which they evolved (Tonnabel *et al.*, 2012; Olivieri *et al.*, 2016; Lamont *et al.*, 2020). Changes in this fire regime may therefore lead to unbalanced population dynamics that hinder seed recruitment in future generations (Nottebrock *et al.*, 2013). Therefore, understanding the life-history dynamics of dioecious Proteaceae can aid and inform guidelines for the effective conservation of the fynbos biome (Schurr *et al.*, 2012).

Previous studies on dioecious Proteaceae have mostly indicated that diverging secondary sex ratios are related to potential differences in reproductive costs. For example, Bond and Maze (1999) observed a female bias (i.e. more females than males) in older (17- to 20-year-old), dense populations of living *Leucadendron xanthoconus*, and suggested that males experience higher reproductive costs due to their extravagant floral displays, which are driven by competition for insect pollinators. By contrast, other researchers have found male-biased sex ratios, suggesting that females experience a higher cost of reproduction (Barrett *et al.*, 2010; Harris and Pannell, 2010). Barrett *et al.* (2010) observed male-biased sex ratios among sexually mature individuals in young (6-year-old) populations and even sex ratios in old (13- to17-year-old) populations of *Leucadendron gandogeri*. Because they observed many individuals in these young populations that did not yet have cones, which they assumed were predominantly immature females, they attributed these patterns of delayed reproductive maturity in females to the costly production of their reproductive structures. Other studies have inferred sex-specific reproductive costs in dioecious Proteaceae by examining traits related to reproductive provisioning, such as serotiny and vegetative growth patterns. In several *Leucadendron* species, Harris and Pannell (2010) found a strong positive association between the degree of serotiny, a proxy for maternal care, and sexual dimorphism in the form of reduced female ramification. They argued that serotinous females incur higher reproductive costs due to the physiological demands of cone maintenance, forcing them to invest fewer resources in vegetative growth. Conversely, Cramer and Midgley (2009) argued that serotiny incurs small reproductive costs in both *Leucadendron* and *Aulax* species.

Recent work by Dorken *et al.* (2025), Pannell (2025) and Dorken (2025) has highlighted misunderstandings (Midgley *et al.*, 2019; Midgley, 2022) regarding the definition of reproductive costs in plants. Midgley *et al.* (2019) argued that the cost of reproduction in dioecious Proteaceae ‘must’ be equal between the sexes, reasoning that equal lifespans imply similar vegetative and reproductive investment. They also argued that co-occurrence with hermaphroditic Proteaceae suggests comparable post-fire average fitness, and the stable coexistence of males and females indicates equal competition for resources. Midgley (2022) further supported this stance in a commentary on Liu *et al.* (2021), by citing equal secondary sex ratios (Midgley and Cramer, 2022) and disputing the claims that vegetative dimorphism implies unequal reproductive allocation. However, Pannell (2025) and Dorken *et al.* (2025) argued against this perspective by emphasizing the distinction between sex allocation theory and life-history theory. They argued that differences in sex ratios do not necessarily imply varying reproductive costs or fitness; such conclusions depend on whether these differences translate into differences in expected future fitness. This ongoing debate, which may partially stem from species-specific selective pressures acting on sexual dynamics (de Jong and Klinkhamer, 2002) or small sample sizes of sex ratio estimates and in turn low statistical power (Wilson and Hardy, 2002), justifies further empirical investigation. Dorken (2025) advocated the incorporation in such studies of both reproductive trade-offs and life-history dynamics to clearly demonstrate that costs of reproduction arise from balancing resource investment in current reproduction against conserving resources for future reproductive opportunities, affecting overall fitness.

To resolve discrepancies between previous studies we investigated several species of dioecious Proteaceae (Supplementary Data Table S1) to determine whether they exhibit sex-specific life-history dynamics and reproductive trade-offs that relate to differences in reproductive costs (Dorken, 2025). Given the fire-adapted ecology of obligate reseeders, which produce single-aged cohorts (Rebelo, 2001), we assessed potential life-history differences or sex-biased mortality by quantifying how secondary sex ratios (de Jong and Klinkhamer, 2002) vary with post-fire age. Specifically, we tested three alternative hypotheses with different predictions for the relationship between adult secondary sex ratio and post-fire age. The higher female cost hypothesis predicts that increased female mortality is due to the resource demands of the physiological costs of cone production and maintenance, resulting in male-biased secondary sex ratios in older populations. Conversely, the higher male cost hypothesis predicts that increased male mortality is due to the resource demands of extravagant floral displays and pollen production, resulting in female-biased secondary sex ratios in older populations. Finally, the equal cost hypothesis states that reproductive costs do not differ between the sexes and predicts consistent equal secondary sex ratios across populations of different post-fire ages. As a further test for diverging life-history trade-offs we assessed overall canopy condition (Schomaker *et al.*, 2007), a proxy for plant health status, in varying-aged stands of *Leucadendron laureolum.* Following recommendations by Dorken (2025), we further assessed potential diverging reproductive trade-offs between the sexes by quantifying sex-related differences in nutrient allocation to reproductive and vegetative structures (Bazzaz *et al.*, 2005) in a population of *L. gandogeri*.

## MATERIALS AND METHODS

### Study system and species

This study was conducted in the fynbos biome in the Western Cape, South Africa, which is a Mediterranean-type ecosystem characterized by cool, wet winters and hot, dry summers (Rebelo, 2001). Proteaceae are a dominant overstorey taxa in the fynbos biome, and in comparison with other co-occurring taxa they are relatively long-lived and slow to reach maturity, making them an ideal species for population and demographic studies (Schurr *et al.*, 2012). The sexes in dioecious Proteaceae genera *Leucadendron* and *Aulax* are easily identifiable through their unique sexual structures (Rebelo, 2001). Females have seed-bearing cones that become hard and woody after flowering and males have smaller pollen-bearing cones on the terminal growing shoots, and in many species the surrounding leaves of both sexes can turn yellow or red during the flowering season to attract pollinators (Barker *et al.*, 2004). These Proteaceae generally take 2–3 years to develop their first cones and then produce one terminal cone per stem, per season (Midgley and Rebelo, 2008). The age of the plant can be estimated by counting the number of branching nodes and adding 3 (Harris and Pannell, 2010). While diverging secondary sex ratios can be attributed to other factors, such as differential clonal expansion, the study species in question only naturally reproduce sexually and therefore one can assume that the diverging ratios in this system are due to sex-differential mortality rates.

### Life-history dynamics: sex ratios

We examined nine species of serotinous Proteaceae across 11 sites (34 species–site populations) covering a broad geographical range within the fynbos biome (Supplementary Data Table S1). The sampled species were obligate reseeders, meaning that they can only reproduce through post-fire seed germination (Barker *et al.*, 2004), except for *L. salignum*, which is capable of resprouting and surviving fire. Therefore *L. salignum* was removed from the overall statistical analysis as it can be older than its branching pattern indicates (Pausas *et al.*, 2004), leaving eight species of obligate reseeding serotinous Proteaceae across 29 species–site populations. Mostly, sites were selected opportunistically and for ease of access, focusing on relatively natural populations in reserves. Effort was made to ensure that a wide range of post-fire ages were sampled, but more common species are more proportionally represented in the sampling. Sex ratios were recorded by counting males and females in a 5-m wide transect, stopping at regular intervals to age individuals and counting until a large enough sample size was reached. This was determined on a case-by-case basis and informed by a pre hoc power analysis using the binomial distribution. In one instance an entire population (*L. teretifolium*) consisting of 80 individuals was sexed. In each population, the ages of individuals were similar, from which we obtained an estimate of the overall population age. The sex ratios were calculated in accordance with Wilson and Hardy (2002) as the proportion of males over the total population so that a female bias is represented as a ratio <0.5 and a male bias as a ratio >0.5. While this technically is not a true ratio but rather a proportion, it is the literature standard, and the term ‘ratio' was therefore used in this study.

To determine whether there was an effect of age on the sex ratios of different populations, a Bayesian multilevel regression model was implemented using the brms package (version 2.21.6; Bürkner, 2017) in the R environment R-4.4.1 (R Core Team, 2025). The sex ratio was modelled with a binomial response distribution using the logit link function, following the recommendations of Wilson and Hardy (2002). The first model included a species-level random intercept term and followed the form male ∣ (male + female) ∼ age + (1 ∣ species), which allows for variability between species intercepts but assumes the effect (slope) of age on the sex ratio is fixed across species. The second was a model with random slopes following the form male ∣ (male + female) ∼ age + (age ∣ species), which is an extension of the first, but additionally allows the effect (i.e. slope) of age to vary between species. Both models additionally included an observation-level random effect as a means of quantifying residual variation. The brms package uses Hamiltonian Monte Carlo (Neal, 2011) for parameter estimation via the Stan probabilistic programming language (Carpenter *et al.*, 2017). Four chains were run, each with 2000 iterations (including 1000 warm-up samples), and adapt_delta was set to a value of 0.99 to enhance sampling efficiency and ensure robust convergence. To determine which model provided the best fit, leave-one-out cross-validation (LOO-CV) was implemented through the loo package by estimating the expected log pointwise predictive density (ELPD) (Gabry *et al.*, 2024). The models used default priors, which are relatively uninformative. Convergence diagnostics, including Gelman–Rubin statistic *Ȓ* values and effective sample size (ESS), were examined to ensure adequate mixing of the Markov chains. While this Bayesian approach is apparently novel in the context of plant sex ratio analysis, it has been previously applied to ant colony sex ratios (Lubertazzi and Adams, 2010) and variations in human sex ratios (Long *et al.*, 2021).

### Life-history dynamics: health analysis

To investigate how life-history dynamics may contribute to deviations in sex ratios, the overall canopy condition was measured by scoring the health of three *L. laureolum* populations in Silvermine Nature Reserve (Supplementary Data Table S1). These populations were strategically selected after the sex ratio count because they represent the same species but varied sequentially in age (4, 8 and 10 years) and correspondingly in sex ratio (0.50, *n* = 619; 0.53, *n* = 247; 0.62, *n* = 316, respectively). The three sites were geographically close (within a 1-km radius), which helped ensure similar environmental conditions and limited variability other than age and sex ratio. For each population 30 each of male and female plants were sampled along a 5-m wide transect. Their basal diameter, total height, and a qualitative health score representing percentage of living canopy cover (Supplementary Data Table S2) were recorded. Scores reflect the percentage of branches with living green leaves, where higher values indicate better canopy condition (plant health) and a score of 0 indicates that the plant was dead.

To model how the health scores varied between the sexes across the three sites, while conditioning on the effect of basal diameter and height, we used the *brms* package to fit a linear ordinal regression model (Bürkner, 2017). This model used the cumulative response distribution (logit link), with scores modelled as a function of plant height, basal diameter, site (i.e. population), sex, and the interaction between site and sex (Bürkner and Vuorre, 2019). Posterior predictions of the probability of each score for each sex at each site, averaging over the effects of basal diameter and height, were obtained using the avg_predictions function from the *marginaleffects* package (Arel-Bundock *et al.*, 2024). The model used default weakly informative priors, and the joint posterior density was sampled with four chains of 2000 iterations each (including 1000 warm-up samples). Convergence diagnostics, including Gelman–Rubin statistic *Ȓ* values and ESS, were examined to ensure adequate mixing of the Markov chains.

### Reproductive trade-offs: nutrient analyses

Potential diverging reproductive trade-offs were assessed through the nutritional composition of reproductive and vegetative tissue in a population of *L. gandogeri* (collected at Betty’s Bay; Supplementary Data Table S1). *L. gandogeri* is a relatively common species and this population was 10 years old, a typical age for mature fynbos (Kraaij and van Wilgen, 2014). The two most recent years’ worth of growth were collected at the beginning of spring from five male and five female plants, when males bore the current season's pollen-bearing inflorescence and females bore cones from the previous flowering season, with no new cones present. While this is asynchronous, it could reflect the hypothesized ongoing nutrient demands of females of serotinous Proteaceae species, which maintain their cones for ∼3 years (Cramer and Midgley, 2009; Harris and Pannell, 2010). For each individual, stem diameters were measured at the base of 2-year-old stems using a calliper to enable calculation of the cross-sectional area subtending the vegetative and reproductive tissue. The cones (reproductive structures) were separated from the stems and leaves (vegetative structures), and all samples were dried at 70 °C for 48 h. After drying, the vegetative and reproductive structures were weighed separately for each sample to obtain the biomass for each structure. The dried samples were then ground using a Wiley mill with a 0.5-mm mesh. The samples were analysed for N, P, K, Ca, Mg, Na, Mn, Fe, Cu, Zn and B content by BemLab (Pty) Ltd. The total leaf N was determined through combusting pulverized leaf material on an FP-528 Nitrogen Analyser (Leco Corporation, St Joseph, USA). The other nutrients, P, K, Ca, Mg, Na, Mn, Fe, Cu, Zn and B, were determined by dry-ashing pulverized leaf material at a temperature of 480 °C for 8 h and dissolving it in HCl (Kalra, 1998). The solution was analysed using inductively coupled plasma atomic emission spectrometry (Varian Vista MPX ICP-AES, Australia). The results were reported as nutrient concentrations in percentages or mg/kg per sample and nutrient concentrations per unit stem cross-sectional area were calculated.

To determine if there was a difference in nutrient allocation between the sexes, the content of nutrients in both reproductive and vegetative structures was calculated, and then the proportion of nutrients in the reproductive structures was expressed relative to the overall content of both structures. Using the emmeans (Lenth, 2023) and multcomp (Hothorn *et al.*, 2008) packages, a one-way ANOVA was conducted to identify statistically significant differences in overall nutrient concentrations between the sexes. This was followed by post hoc comparisons with Tukey’s honest significant difference (HSD) test to determine pairwise differences. To determine whether there was a difference between the sexes in the proportion of nutrients allocated to reproductive and vegetative structures, a beta regression model was implemented using the betareg package (Zeileis *et al.*, 2024). This model accounts for the bounded nature of the nutrient proportion data and provides both the mean and dispersion of the nutrients allocated to reproductive structures (Geissinger *et al.*, 2022), providing insight into potential differences in both average allocation to reproductive structures and its variability between the sexes.

## RESULTS

### Life-history dynamics: sex ratios

**Fig. 1.**
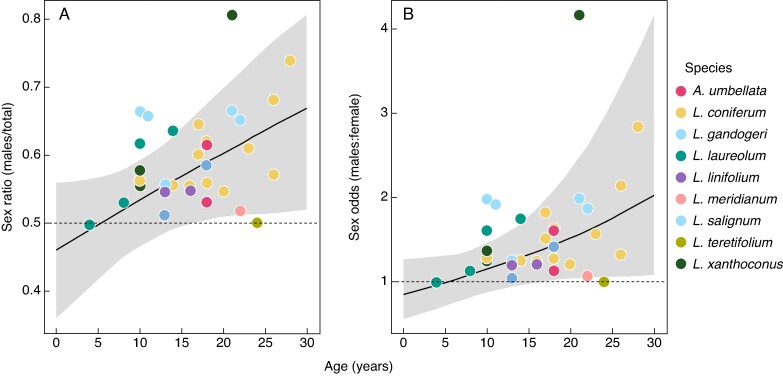
The relationship between population age and sex across species (*n* = 8) of dioecious Proteaceae. A Bayesian random slope model was implemented, with species and population as random effects. While the model did not include *L. salignum* as it is a resprouter, it was plotted to emphasize that it is still male-biased. Shaded areas represent the 95 % credible intervals. (A) Sex ratio as calculated by the proportion of males over total (males/males + females) plotted against population age. The dashed line indicates an even proportion. (B) Same model predictions shown on an odds scale (sex ratio = *P*/[1 − *P*]) against population age. Again, the dashed line indicates an even sex ratio (1:1).

A total of 29 populations of seven species of *Leucadendron* and one species of *Aulax* were sampled across the Western Cape in varying climate conditions and soil types (Supplementary Data Table S1). The majority of populations were male-biased (mean sex ratio = 0.59, minimum = 0.50, maximum = 0.81; Fig. 1). Model diagnostics showed that both models demonstrated strong statistical performance. Bulk ESS and tail ESS exceeded 1000, ensuring sufficient independent samples for reliable estimation. The LOO-CV results indicated that the random slope model slightly outperformed the random intercept model (ELPD difference = 1.5 ± 1.5 s.e.).

The random slope model (Table 1), which excluded the resprouter *L. salignum*, indicated a positive association between male bias and age, predicting that for each additional year of age the ratio of male to female individuals is multiplied by a factor of e^0.029^ = 1.029 on average. The random slope model predicts that an average 30-year-old population would have a sex ratio of 0.67, corresponding to two males for every one female (Supplementary Data Table S3). However, the species-level variation for age slopes (s.d. = 0.028, 95 % CI 0.008, 0.070) supports modest interspecific differences in age-related sex ratio shifts, suggesting that some species may exhibit a stronger age-related increase in male bias. Additionally, the species-level intercept variation (s.d. = 0.216, 95% CI 0.013, 0.784) was the largest among the random effects, highlighting substantial differences in baseline sex ratios among species. The residual variation at the population level (s.d. = 0.100, 95 % CI 0.021, 0.200) indicates additionally unexplained variation in sex ratio beyond species and age. The random slope model had an intercept of 0.46 (95 % CI 0.36, 0.56) on the sex ratio scale.

**Table 1.** Summary of the Bayesian random slope model for sex ratio as a function of age in obligate reseeding dioecious Proteaceae within the fynbos biome.

	Estimate
Intercept	−0.165 (−0.608, 0.225)
Age	0.029 (−0.001, 0.061)
Species intercept	0.216 (0.013, 0.784)
Species age	0.028 (0.008, 0.070)
Population (residual)	0.100 (0.021, 0.200)
*R* ^2^	0.992
*R* ^2^ marginal mean.	0.971

The model includes species-specific random intercepts and slopes for age, as well as a population-level residual term. Estimates are posterior means with 95 % credible intervals in brackets. Observation level random effects were included to account for residual variation.

### Life-history dynamics: health analysis

**Fig. 2.**
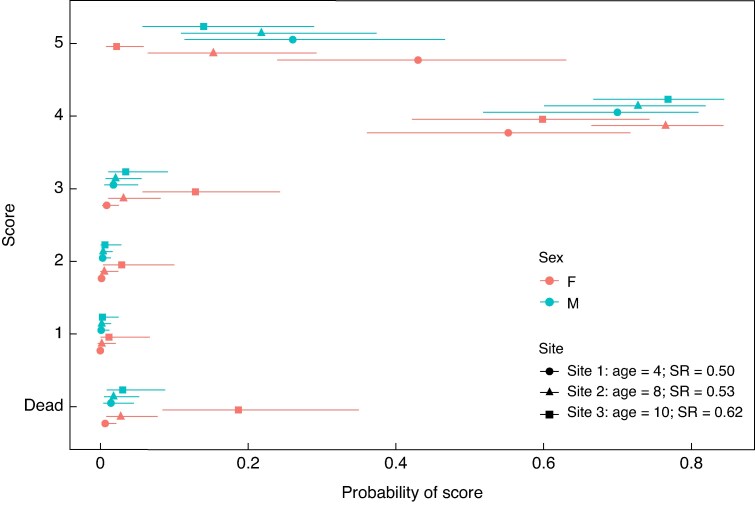
Predicted probabilities of health scores across sex and site for dioecious Proteaceae. Health score data were collected on three populations of *Leucadendron laureolum* at Silvermine Nature Reserve in the Western Cape. Predictions are based on a Bayesian ordinal regression model. The points represent posterior mean probabilities of each sex at each site, with error bars representing the 95 % credible intervals. SR, sex ratio.

The overall health score of the three populations of *L. laureolum* showed that females in the older, male biased populations (site 3: age = 10, sex ratio = 0.62) had lower scores while there was no difference in score between males and females in the younger populations (site 1: age = 4, sex ratio = 0.50 and site 2: age = 8, sex ratio = 0.53) with a sex ratio that did not differ significantly from 0.5. The ordinal regression model showed no significant effects of basal diameter or height on plant health scores across the sites. Additionally, there was no substantial main effect of sex on score across all three sites. However, including an interaction between site and sex revealed notable site-specific differences in health scores between males and females (slope estimate = −2.87, 95 % CI −4.59, −1.26). This indicates that at site three, the oldest and most male-biased, males had a significantly higher probability of attaining a higher health score than females, while females were more likely to have low scores (Fig. 2). The model diagnostics showed strong convergence and stability. The bulk and tail ESS values were >1000, indicating sufficient samples for accurate estimation. Additionally, the *Ȓ* values were all equal to 1.00, confirming that the model chains mixed well and converged properly.

### Reproductive trade-offs: nutrient analysis

**Fig. 3.**
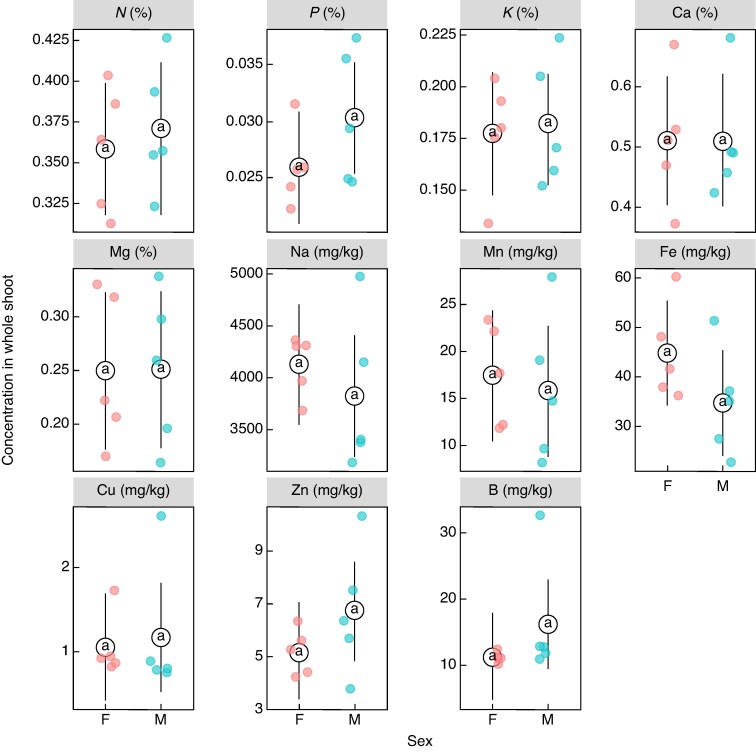
Estimated mean concentration of nutrients in the whole shoot of male (*n* = 5) and female (*n* = 5) plants of *Leucadendron gandogerii* sampled near Betty’s Bay in the Western Cape. The points and error bars represent estimated marginal means derived from an ANOVA. Different letters indicate post hoc pairwise comparison groupings, groups sharing the same letter show no statisitcally significant difference. No significant differences were detected between the sexes for any of the nutrients.

**Fig. 4.**
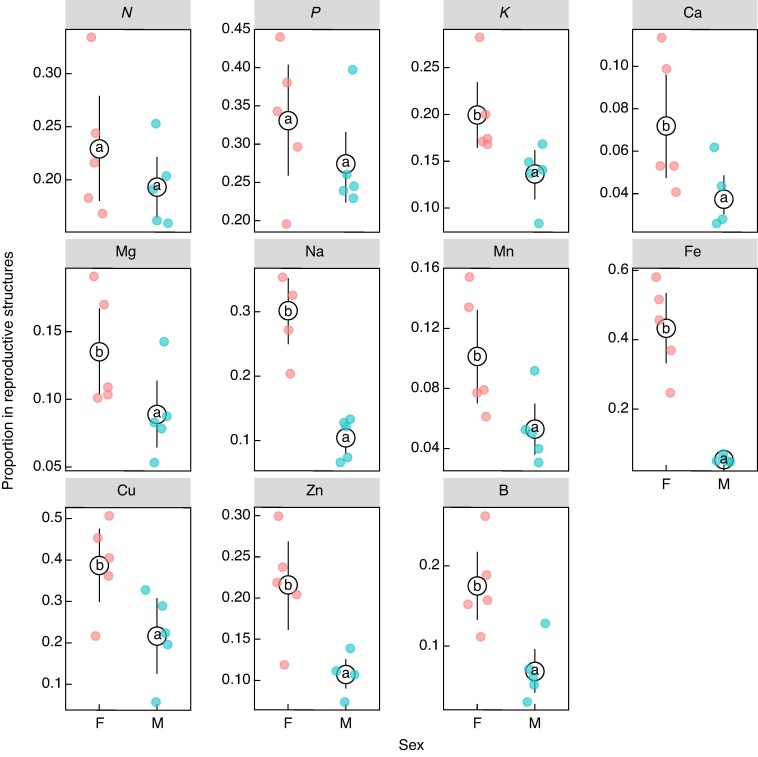
Estimated mean proportion of nutrients allocated to reproductive structures in male (*n* = 5) and female (*n* = 5) plants of *Leucadendron gandogeri* sampled near Betty’s Bay in the Western Cape. Estimates are based on a beta regression model. Points and error bars represent estimated marginal means and standard error for each sex. Different letters indicate post hoc pairwise comparison groupings, groups sharing the same letter show no statistically significant difference and groups with different letters differ significantly. Significant differences between the sexes were found for K, Ca, Mg, Na, Mn, Fe, Cu, Zn and B.

Females allocated a greater proportion of their biomass to reproductive structures compared with males (Supplementary Data Fig. S1). The nutrient concentrations across all components of male and female vegetative and reproductive structures showed that male reproductive structures had higher concentrations of N, P, K and Cu, but lower Ca, while female cones had higher concentrations of P, Na and Fe, and lower Ca, Mg and Mn (Supplementary Data Fig. S2). No differences were observed in vegetative nutrient concentrations between the sexes. The one-way ANOVA showed that across the whole shoot (i.e. both vegetative and reproductive structures) there was no significant difference in the overall concentration of nutrients in the whole shoot (i.e. reproductive and vegetative components) between the sexes (Fig. 3). Additionally, when concentration was expressed per unit cross-sectional area of stem tissue there were no significant differences in the concentration of nutrients between the sexes (data not shown). However, when still accounting for this greater proportion in biomass, the beta regression model showed that the proportion of nutrient allocated to reproductive structures relative to the whole shoot was greater in females than in males (Fig. 4), specifically, in K, Ca, Mg, Na, Mn, Fe, Cu, Zn and B. Notably there was no difference in allocation of N and P between the sexes.

## DISCUSSION

We present evidence of diverging life-histories in several species of dioecious fynbos Proteaceae, which exhibit increasingly male-biased sex ratios and decreasing female vitality in older populations. We further document differing reproductive trade-offs, where females allocate a greater proportion of nutrients to their reproductive structures. Together, these results support the hypothesis that female dioecious Proteaceae experience a higher cost of reproduction than males (Dorken, 2025).

To demonstrate differences in reproductive costs between sexes, one must show differing survival rates resulting from diverging life-history strategies and reproductive trade-offs (Dorken, 2025). Our model showed that male bias increased with population age, since at recruitment (age 0) the expected sex ratio would be 0.46, and in a 30-year-old population it would be 0.67, equivalent to two males for every female (Supplementary Data Table S3). This reflects a pattern of age-related female mortality likely driven by a decline in female vitality, as supported by the health analysis. These greater reproductive costs in females are most likely due to the cumulative maintenance cost associated with serotinous cones (Harris and Pannell, 2010). Additionally, the nutrient analyses support the reproductive costs associated with serotiny and show differing reproductive trade-offs between the sexes. While males and females have equal amounts of nutrients in the whole shoot, including both reproductive and vegetative shoots (Fig. 3), females allocate a greater proportion of their total nutrient content to their reproductive structures (Fig. 4). Diversion of essential nutrients (including K, Ca, Mg, Na, Mn, Fe, Cu, Zn and B) into female reproduction likely comes at the expensive of vegetative maintenance and future growth, aligning with the classic cost of reproduction definition (Tuomi *et al.*, 1983; Obeso, 2002; Dorken *et al.*, 2025). Although no difference in N and P allocation to reproductive structures between the sexes was observed, this is most likely because the fynbos is already so severely limited in these nutrients that Proteaceae have evolved mechanisms to conserve and minimize their N and P requirements (Cramer *et al.*, 2014). The deficiencies of the other essential nutrients in female vegetative structures could, however, be enough to compromise overall plant function and result in the observed male-biased populations. These results support the view that heavier female reproductive investment imposes greater long-term costs on ‘performance’ (e.g. reduced canopy condition and survival) than male investment. This aligns with classic cost-of-reproduction theory, whereby a higher reproductive effort reduces future growth and survival (Tuomi *et al.*, 1983; Obeso, 2002; Dorken *et al.*, 2025), helping to explain the observed sex-biased decline in female vitality and survival and contributing to increasingly male-biased secondary sex ratios in older populations.

We observed substantial variation between species in the effect of age on sex ratios, which is likely driven by physiological and ecological traits specific to each species. The removed *L. salignum* was the only resprouting species sampled in this study (Rebelo, 2001), which may partially explain its high sex ratios. As species capable of resprouting retain individuals across fire events, the current observed sex ratio may reflect a longer history of biased survival compared with reseeding species. For example, the youngest population of *L. salignum* sampled was 10 years post-fire and exhibited a sex ratio of 0.66, which was higher than for non-resprouting species of similar age. Nevertheless, assuming that the plants in these populations are several fire cycles old, their sex ratios are not as high as our model might predict by extrapolation. This could be explained by certain fundamental differences between resprouters and obligate reseeders, such as increased allocation to underground structures, improved capacity to exploit post-fire resources, and alleviation of the female reproductive burden by removal of cones during fires (Cramer *et al.*, 2014). We also observed some cases in which sex ratios of obligate reseeders are unbiased despite old age. For example, two 22-year-old populations of *L. meridianum* both had a sex ratio of 0.52. This species occurs on relatively nutrient rich limestone-derived soils (Rebelo, 2001), which may buffer female individuals from the higher reproductive costs associated with cone production. The observed sex ratio in *L. teretifolium* (0.50) may be less informative due to small sample size (*n* = 80), as the entire local population consists of only 80 individuals. Furthermore, the residual variation at the population level suggests that, even within species, sex ratios are influenced by local-scale factors that were not accounted for in this study. These may include environmental variation, such as local-scale soil nutrient availability or varied microclimates. In addition, demographic stochasticity may lead to random deviations in sex ratios due to chance differences in recruitment or mortality (Lande *et al.*, 2003). This variation reveals that there are species- and site-specific dynamics that shape the sex ratio outcome as local conditions act alongside species traits to influence demographic structure.

The previous studies that used sex ratios to investigate the cost of reproduction in Proteaceae also used them in conjunction with measurements of population density (Bond and Maze, 1999) and allocation to growth (Midgley and Cramer, 2022). Our study did not assess these factors, which may partly explain some of the disparities, although another possible explanation is that the small sample size and limited number of populations surveyed in previous studies reduced their statistical power. When assessing whether an observed sex ratio deviates from 0.5, small sample sizes produce wide uncertainty intervals. Midgley and Cramer (2022) reported sex ratios of 0.45, 0.46, 0.48, 0.44 and 0.50 with sample sizes of 105, 105, 102, 115 and 325, respectively (Supplementary Data Fig. S3). Bond and Maze (1999) reported sex ratios of 0.58, 0.53 and 0.47 for living individuals with sample sizes of 70, 293 and 136, respectively (Supplementary Data Fig. S3). By contrast, our study offers a more detailed perspective of sex ratio dynamics in dioecious Proteaceae, based on more extensive sampling of individuals and sites (*n* = 34) across multiple species (*n* = 9) and a broader geographic range. Nevertheless, our sample is by no means comprehensive. For instance, many *Leucadendron* species are non-serotinous, dropping their seeds shortly after the cones reach maturity and exhibiting continuous recruitment (Rebelo, 2001). As we were interested in the influence of the fire cycle on the possibility of the existence of strongly diverging sex ratios, we did not include any such species, but further investigation to see if females still incur greater reproductive costs in the absence of serotiny would be interesting.

In summary, our results support a greater reproductive cost in female Proteaceae, who allocate more heavily towards current reproduction, resulting in a trade-off between immediate reproductive success and long-term survival, ultimately reducing their chances of future reproduction. These greater costs present as increased female mortality and are predominantly realized in older populations. This could have conservation implications, particularly in areas experiencing altered fire regimes through anthropogenic fire suppression (Slingsby *et al.*, 2020). Serotiny is an adaptive reproductive strategy that is only viable when the fire return interval remains within a moderate, evolutionarily stable range (Lamont *et al.*, 2020). However, when anthropogenic activities extend the fire return interval beyond this range, serotinous non-resprouting species are thrust into a senescence risk scenario in which serotiny becomes maladaptive (Lamont *et al.*, 2020). With females having higher reproductive cost, greater mortality and a higher rate of senescence, it could compromise future recruitment potential. Indeed, premature loss of reproductive females has been shown to reduce seed production in *L. rubrum* (Nottebrock *et al.*, 2013), which may reduce recruitment and genetic diversity in future generations (Rosche *et al.*, 2018). Such outcomes could have implications for the long-term viability of dioecious Proteaceae species in the fynbos biome if fire suppression persists. Our findings further highlight how future fire management strategies and policies should also account for species’ evolutionary history (Olivieri *et al.*, 2016).

## Supplementary Material

mcaf312_Supplementary_Data

## SUPPLEMENTARY DATA

Supplementary data are available at *Annals of Botany* online and consist of the following. Table S1: populations of dioecious Proteaceae sampled across the Western Cape. Table S2: description of Likert scale used to score percentage of living canopy cover for the health analysis of three populations of *Leucadendron laureolum* in Silvermine Nature Reserve. Table S3: predicted male-to-female ratios across age classes, calculated using the Bayesian random slope model. Figure S1: biomass allocation to vegetative and reproductive structures in male (blue) and female (red) individuals of *Leucadendron gandogeri*. (A) Total mass of each individual. (B) Proportion of mass for each individual. Figure S2: nutrient concentration in male and female vegetative and reproductive tissue sampled of *Leucadendron gandogeri*. Figure S3: credible intervals (95 %) for sex ratio estimates across a range of sample sizes, under the null hypothesis of a 0.5 sex ratio.
