# Supplementary material for: Diverging sex ratios in dioecious Proteaceae are exacerbated by anthropogenic disruptions to the fire cycle
Source: Ann Bot. 2025 Dec 9;137(5):1169–78. doi: 10.1093/aob/mcaf312 (PMC13197573; doi:10.1093/aob/mcaf312)
Supplement: mcaf312_Supplementary_Data [file mcaf312_supplementary_data.pdf]

Supplementary Material

Table S1: Populations of dioecious Proteaceae sampled across the Western Cape. Includes species identity, population age (years since fire), observed sex ratio (males/total) with 95% confidence intervals, total number of individuals sampled, and geographic co-ordinates (latitude and longitude) for each population.

Site	Species	Years Post-Fire	Obs. SR [95% CI]	Total	Latitude	Longitude
Betty's bay	*L. gandogeri*	13	0.51 [0.48,0.55]	767	-34.35099	18.90881
	L. xanthoconus	10	0.55 [0.5,0.6]	346	-34.35099	18.90881
Brodylink	*L. coniferum*	28	0.74 [0.53,0.87]	23	-34.36451	18.86350
	L. coniferum	26	0.68 [0.58,0.77]	107	-34.36389	18.86266
Cape Point	*L. coniferum*	26	0.57 [0.51,0.63]	265	-34.30638	18.43528
	L. laureolum	14	0.64 [0.57,0.71]	187	-34.29200	18.44489
Fairy glen	*L. coniferum*	10	0.56 [0.51,0.62]	334	-34.32896	18.99528
Flower Valley	*L. coniferum*	18	0.62 [0.58,0.65]	713	-34.54676	19.46273
Grootbos	*L. coniferum*	17	0.6 [0.53,0.67]	168	-34.52992	19.49213
	L. coniferum	17	0.65 [0.57,0.72]	141	-34.53282	19.48933
	L. coniferum	18	0.56 [0.52,0.6]	573	-34.52414	19.45250
	L. coniferum	16	0.56 [0.51,0.6]	445	-34.52885	19.46619
	L. coniferum	14	0.56 [0.51,0.6]	450	-34.54964	19.42515
	L. coniferum	20	0.55 [0.51,0.58]	778	-34.54412	19.43053
Kleinmond	*A. umbellata*	13	0.55 [0.5,0.6]	367	-34.32982	19.03336
Palmiet Riv	*L. gandogeri*	18	0.59 [0.52,0.64]	280	-34.33326	18.99203
Platbos Esc	*L. coniferum*	23	0.61 [0.56,0.66]	359	-34.56709	19.43630
Potberg	*A. umbellata*	18	0.62 [0.56,0.67]	317	-34.38614	20.54224
	A. umbellata	18	0.53 [0.47,0.59]	294	-34.40020	20.55643
	L. linifolium	13	0.55 [0.49,0.6]	363	-34.41138	20.58053
	L. linifolium	16	0.55 [0.51,0.59]	511	-34.40517	20.56416
	L. meridianum	22	0.52 [0.46,0.57]	378	-34.42339	20.41187

Site	Species	Years Post-Fire	Obs. SR [95% CI]	Total	Latitude	Longitude
	L. meridianum	22	0.52 [0.46,0.57]	321	-34.44098	20.41940
	L. salignum	21	0.67 [0.59,0.73]	206	-34.38614	20.54224
	L. salignum	10	0.66 [0.62,0.71]	464	-34.40020	20.55643
	L. salignum	13	0.56 [0.47,0.64]	140	-34.41138	20.58053
	L. salignum	11	0.66 [0.6,0.71]	283	-34.40976	20.57907
	L. salignum	22	0.65 [0.6,0.7]	416	-34.39794	20.54980
	L. teretifolium	24	0.5 [0.39,0.61]	80	-34.37136	20.52428
	L. xanthoconus	21	0.81 [0.74,0.86]	160	-34.40095	20.55342
	L. xanthoconus	10	0.58 [0.52,0.63]	270	-34.40585	20.56533
Silvermine	*L. laureolum*	8	0.53 [0.47,0.59]	247	-34.09290	18.42302
	L. laureolum	4	0.5 [0.46,0.54]	619	-34.09383	18.42400
	L. laureolum	10	0.62 [0.56,0.67]	316	-34.09154	18.42398

Table S2: Description of Likert Scale used to score percentage of living canopy cover for the health analysis of three populations of *Leucadendron laureolum* in Silvermine Nature Reserve. Scores reflect the percentage of branches with living green leaves, where higher values indicating better plant health.

Scores	Percentage of canopy cover (%)	Description
0	0	Dead with no green leaves
1	1-25	1-25% of the branches contain living green leaves.
2	26-50	26-50% of the branches contain living green leaves.
3	51-75	51-75% of the branches contain living green leaves.
4	76-99	76-99% of the branches contain living green leaves but no new growth.
5	100	Healthy representation of new growth and flowers (season dependant).

Table S3: Predicted male-to-female ratios across age classes, calculated using the Bayesian random slope model. The Sex Ratio is calculated as the number of males divided by number of males plus number of females. The Sex Odds is calculated as sex ratio/(1-sex ratio). Values represent posterior means with 95% credible intervals in brackets.

Age	Proportion (M/Total)	Ratio (M:F)
0	0.46 [0.36, 0.56]	0.85:1 [0.56:1, 1.3:1]
10	0.53 [0.47, 0.59]	1.1:1 [0.88:1, 1.4:1]
20	0.6 [0.51, 0.7]	1.5:1 [1:1, 2.3:1]
30	0.67 [0.52, 0.81]	2:1 [1.1:1, 4.2:1]

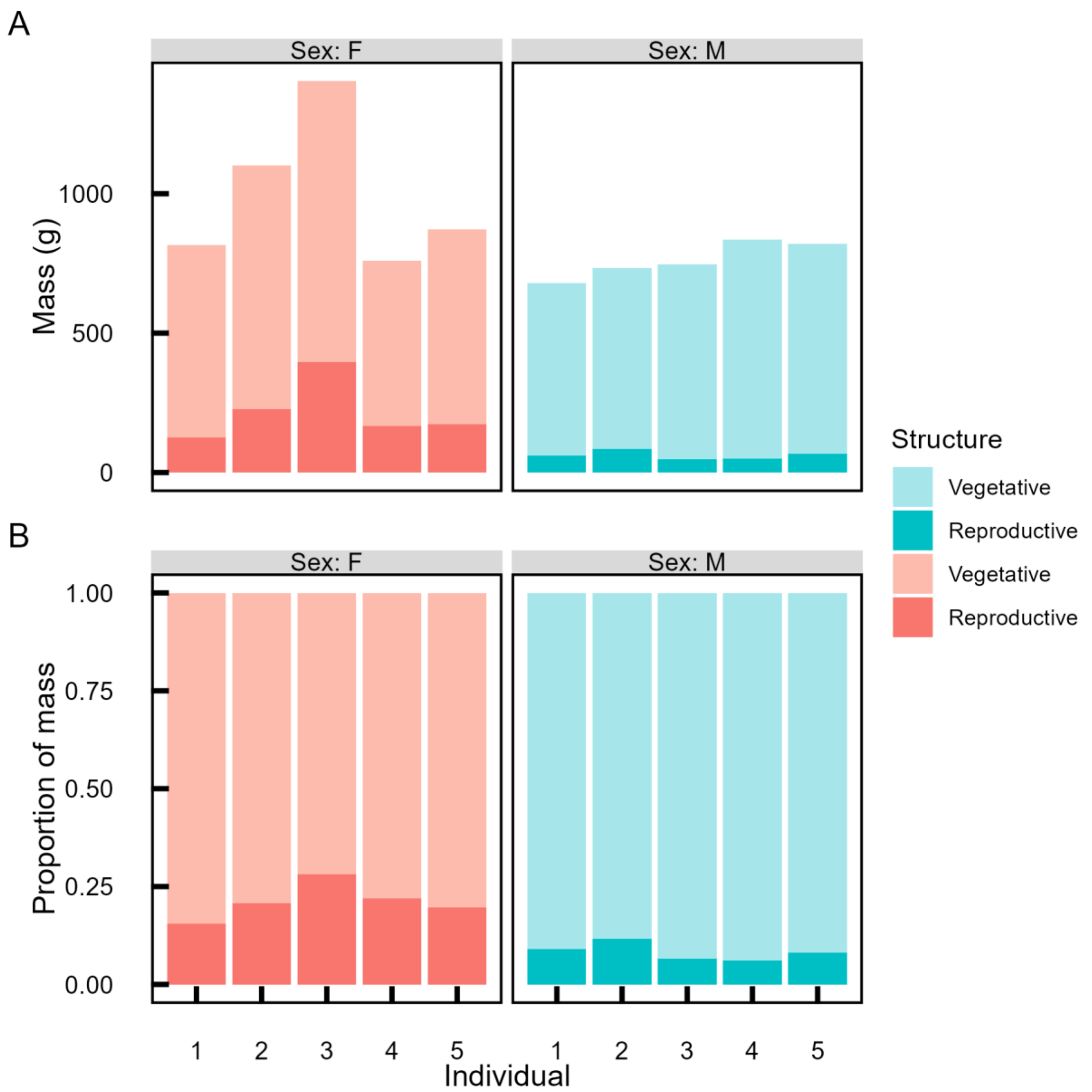

Figure S1: Biomass allocation to vegetative and reproductive structures in male (blue) and female (red) individuals of *Leucadendron gandogeri.* A) Total mass of each individual. B) Proportion of mass for each individual.

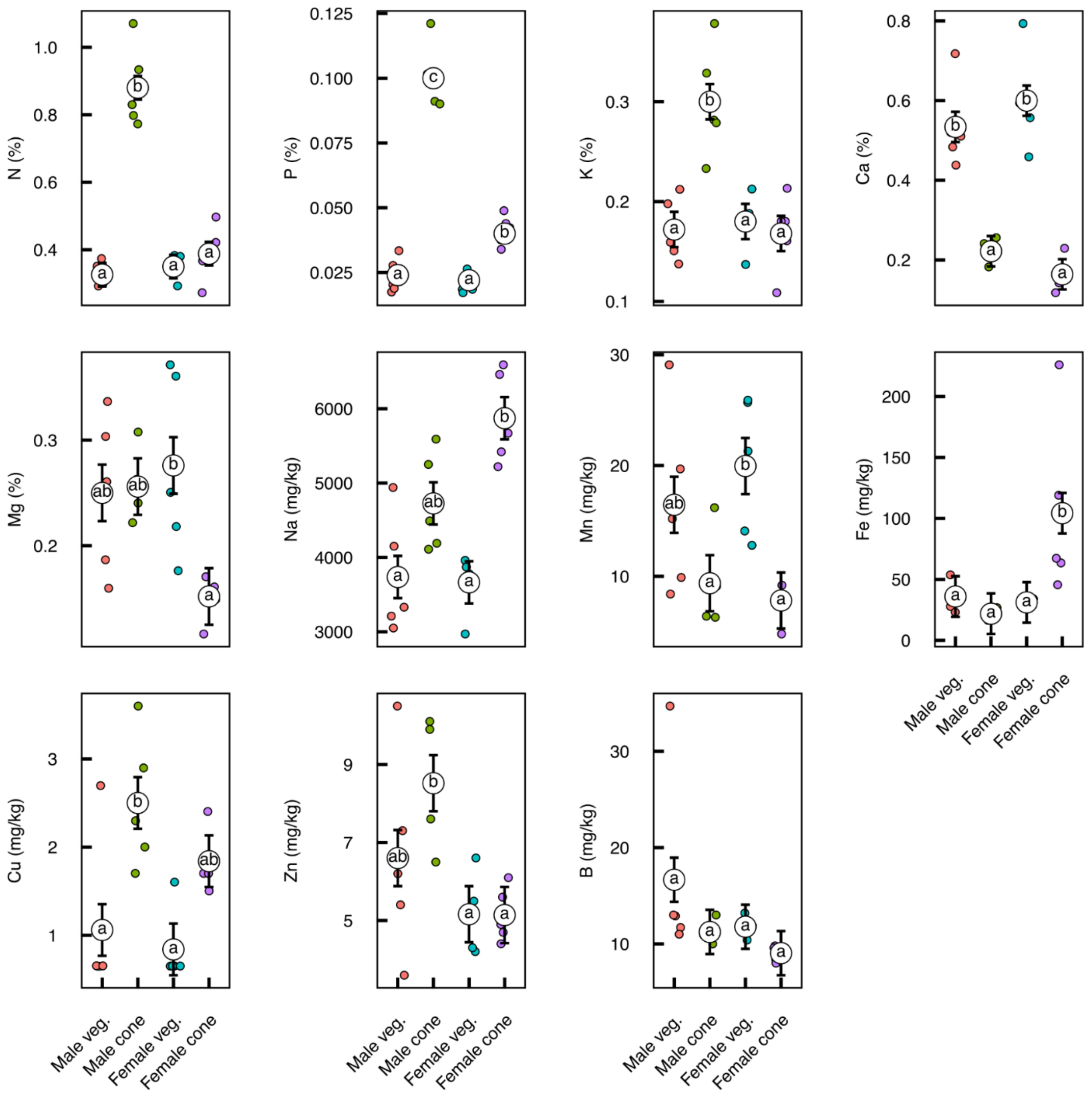

Figure S2: The nutrient concentration in male and female vegetative and reproductive tissue sampled of *Leucadendron gandogeri*. The points represent concentrations in individual plants, and the open circles indicate the means ± SE with compact letter display indicating significant pairwise differences (Tukey HSD, p = 0.05).

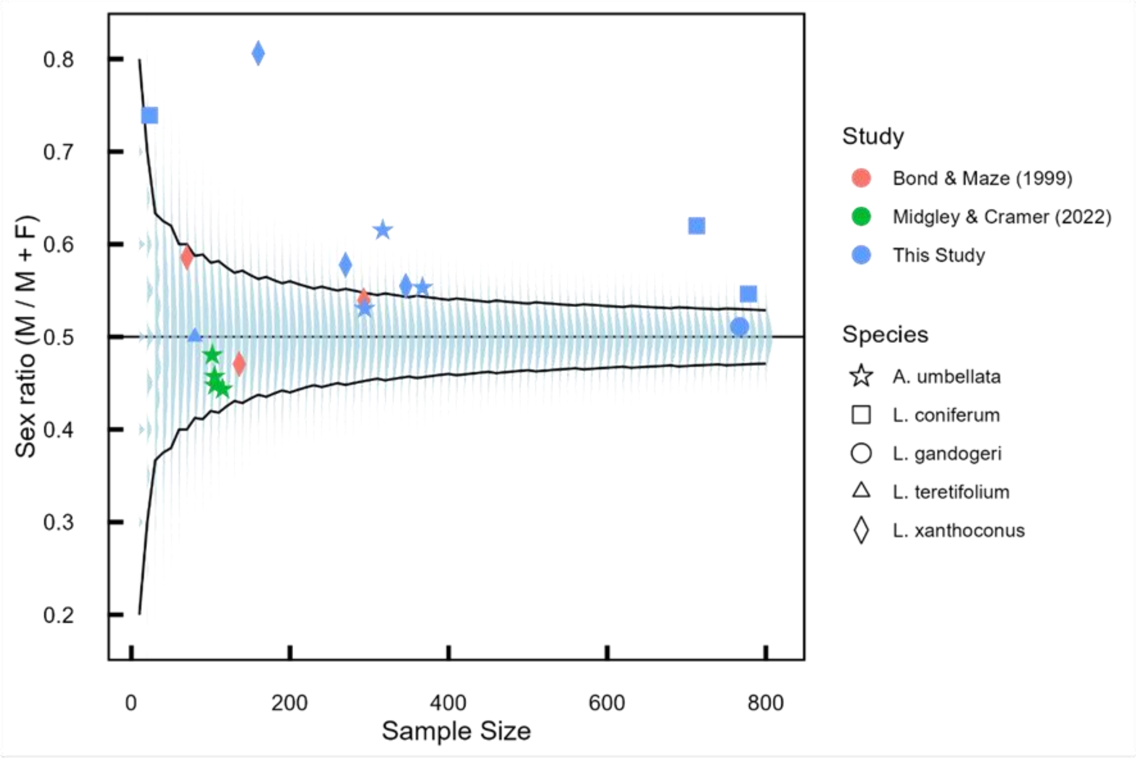

Figure S3: Credible intervals (95%) for sex ratio estimates across a range of sample sizes, under the null hypothesis of a 0.5 sex ratio. The curve represents 95% credible intervals for sex ratio estimates assuming a balanced sex ratio. Overlaid points include sex ratio observations from previous studies of dioecious Proteaceae, corresponding species data from this study (blue), and additional data point from this study that emphasize the effect of sample size on credible intervals. Additionally, points falling outside the credible interval curve indicate statistically significant ($p < 0.05$) deviation from a balanced sex ratio.
